# Decreased susceptibility to chlorhexidine and distribution of qacA/B genes among coagulase-negative *Staphylococcus* clinical samples

**DOI:** 10.1186/s12879-019-3823-8

**Published:** 2019-02-27

**Authors:** Bruna Costa Moura do Vale, Acácia Gentil Nogueira, Thiago André Cidral, Maria Carolina Soares Lopes, Maria Celeste Nunes de Melo

**Affiliations:** Department of Microbiology and Parasitology, Medical Bacteriology Laboratory, Bioscience Center, Federal University of Rio Grande do Norte – UFRN, Av. Senador Salgado Filho, S/N, Campus Universitário, Lagoa Nova, Natal, RN 59072-970 Brazil

**Keywords:** Coagulase-negative *Staphylococci*, Chlorhexidine, Healthcare-associated infection, *qacA/B* genes

## Abstract

**Background:**

Healthcare-associated infection (HAI) is a major public health problem. As a form of prevention and control, preparations of chlorhexidine are used extensively; however, the reduction of susceptibility to chlorhexidine has been reported. The aim of this study was to investigate the susceptibility to chlorhexidine and the distribution of the *qacA/B* genes in 211 clinical isolates of coagulase-negative Staphylococci (CoNS).

**Methods:**

CoNS were identified by conventional biochemical tests. Antimicrobial susceptibility was tested by disk-diffusion. Minimum inhibitory concentration (MIC) of chlorhexidine was determined by agar dilution test; detection of the *qacA/B* and *mecA* genes were evaluated by PCR.

**Results:**

The most frequently isolated species were *S. epidermidis*, *S. hominis hominis*, *S. auricularis,* and *S. haemolyticus*, respectively. The strains presented a multidrug resistance profile of 87%, including methicillin resistance. Reduced susceptibility to chlorhexidine was observed in 31%. *The qacA/B* genes were detected in samples resistant (32/32) and susceptible (17/32) to chlorhexidine. The vast majority (94%) of the samples with reduced susceptibility to chlorhexidine were multidrug resistant.

**Conclusions:**

Our results show that *qacA/B* genes are not restricted to strains expressing chlorhexidine resistance. Further studies are needed to understand how the expression of these genes occurs.

## Background

Healthcare-associated infections (HAIs) have become a serious problem for global public health [1]. Coagulase-negative Staphylococci (CoNS) are among the main microorganisms involved in HAIs, particularly bloodstream infections and infections related to the use of invasive medical devices [2]. To prevent the occurrence of HAIs, control measures are required, such as the rational use of antibiotics and antiseptics. Chlorhexidine is the most commonly used antiseptic in healthcare settings in infection control programs [3, 4] and is a cationic bisbiguanide used for different purposes such as in the decolonization of surfaces, in the treatment of puncture sites of central venous catheters, for washing patients in intensive care units, for the preoperative treatment of skin, and in hand hygiene [5]. Its mechanism of action involves damage in the outer cell wall layers and the cytoplasmic membrane causing the extravasation of intracellular constituents and consequently the destruction of the bacterium [6]. In recent years, there have been reports of the emergence of clinical isolates of *Staphylococcus* with reduced susceptibility to chlorhexidine, being mainly attributed the presence of the genes *qacA* and *qacB*, which are located in plasmids and are very closely related. Therefore, the polymerase chain reaction (PCR) products are designated as *qacA/B* positive or negative [7]. Their functions consist of coding the proton-dependent efflux pump (QacA/QacB), a member of the major facilitator superfamily of transport proteins, organized into 14 α-helical transmembrane segments, capable of exporting chlorhexidine out of the bacteria. Some studies have suggested cross-resistance between the *qacA/B* genes and other antimicrobial agents, as the plasmids may carry multiple determinants of antimicrobial resistance, facilitating the maintenance of the strains in the hospital environment and their dissemination [8–10].

The presence of antiseptic resistance genes in clinical isolates of *Staphylococcus aureus* and CoNS is considered a novel issue in the practice of infection control. It is necessary to clarify some relevant points regarding the expression of this resistance, especially in CoNS [11, 12].

The objective of the present study was to investigate the frequency of isolates with reduced susceptibility to chlorhexidine and to evaluate the distribution of the *qacA/B* genes in clinical samples of coagulase-negative Staphylococci.

## Methods

### Bacterial strains

A total of 211 CoNS clinical isolates from six public and private hospitals in the city of Natal - RN, Brazil, collected from 2010 to 2015 were analysed in this study. The distribution of collection sites was as follows: 145 (69%) blood, 31 (15%) secretions, 21 (10%) catheter tip, 7 (3%) biological fluids, and 7 (3%) clinical specimens classified as others. The isolates were identified at the species level through conventional biochemical tests [13].

### Antimicrobial susceptibility

Antibiotic susceptibility testing was performed via the disk diffusion method in accordance with the Clinical and Laboratory Standards Institute (CLSI) Guidelines [14]. The antibiotics tested were penicillin G (Pen, 10 U), cefoxitin (Fox, 30 μg), linezolid (Lzd, 30 μg), erythromycin (Ery, 15 μg), gentamicin (Gen, 10 μg), clindamycin (Cli, 02 μg), tetracycline (Tet, 30 μg), ciprofloxacin (Cip, 05 μg), rifampicin (Rif, 05 μg), chloramphenicol (Chl, 30 μg), and sulfamethoxazole/trimethoprim (Sxt, 25 μg). A doubledisk diffusion test (D-test) was also performed to detect induced resistance to clindamycin. The antibiotic disks were purchased from Newprov (Paraná, Brazil). Vancomycin susceptibility was determined with the Etest according to the manufacturer’s instructions (Liofilchem, Teramo, Italy). Oxacillin resistance was confirmed by detection of the *mecA* gene by standard PCR using primers and conditions previously described [15]. The isolates that showed resistance to three or more different classes of antimicrobials were considered multidrug resistant (MDR) [6].

### Minimum inhibitory concentration determination

The determination of the Minimum Inhibitory Concentration (MIC) for chlorhexidine was performed using the agar dilution method [14]. The isolate was considered resistant to chlorhexidine when the MIC was ≥4 mg/L [9, 16, 17]. Seven different concentrations were tested by twofold dilution (0.125 - 8 mg/L), prepared from a stock solution of chlorhexidine of 800 mg/L. Plates were incubated at 35 °C and the reading was performed after 24 h.

### Detection of the *qacA/B* genes

To investigate the presence of the *qacA/B* genes, 32 samples with high MIC (4-8 mg/L) and 32 samples with MIC of 0,125 – 2 mg/L were randomly selected. The genomic DNA was extracted following the thermal lysis methodology as previously described [18]. Conventional PCR was performed to detect the *qacA/B* genes, with the following sets of primers: 59-GCAGAAAGTGCAGAGTTCG-39 and 59-CCAGTCCAATCATGCCTG-39 (product size 361 bp) [19]. 5 μl DNA (100 ng) was added into the PCR mixture containing 12.5 μl 1X PCR Master Mix (dNTP, MgCl2, and Taq DNA polymerase; Promega, USA), 5.5 μl nuclease-free water, and 1 μl (0.4 mM) of each primer. The PCR conditions used for detection of genes *qacA/B* were as follows: initial denaturation step of 96 °C for 3 min, 25 cycles of 94 °C for 20 s, 53 °C for 20 s, and 72 °C for 20 s, and a final extension step at 72 °C for 5 min. PCR products were analyzed by agarose gel electrophoresis. *S. aureus* 7644 strain was used as positive control and nuclease-free water as negative control.

## Results

### Bacterial strains

A total of 211 clinical samples of coagulase-negative Staphylococci were analyzed. It was possible to identify 14 different species of coagulase-negative *Staphylococci*, where the most frequently isolated species were *S. epidermidis*, *S. hominis hominis*, *S auricularis,* and *S. haemolyticus*, respectively. Twenty-eight samples had inconclusive identification and were, therefore, classified only as coagulase-negative *Staphylococcus* (Table 1).

**Table 1:** Multidrug resistance, resistance to chlorhexidine and resistance to methicillin from 211 clinical isolates of CoNS by species

Species	n (%)	MDR n (%)	MIC of CHX ≥ 4 mg/L n (%)	MIC of CHX ≥ 4 mg/L and MR n (%)
*S. epidermidis*	65 (31)	54 (83)	25 (38.4)	22 (33,8)
*S. hominis hominis*	32 (15.1)	26 (81)	10 (31.2)	10 (31,2)
*S. auricularis*	25 (12)	24 (96)	13 (52)	11 (44)
*S. haemolyticus*	24 (11.3)	21 (87.5)	8 (33.3)	8 (33,3)
*S. capitis urealyticus*	11 (5.2)	10 (91)	4 (36.3)	4 (36,3)
*S. saccharolyticus*	5 (2.3)	4 (80)	1 (20)	1 (20)
*S. chonii urealyticum*	5 (2.3)	5 (100)	0	0
*S. cohnii cohnii*	4 (1.8)	4 (100)	0	0
*S. saprophyticus*	4 (1.8)	4 (100)	1 (25)	1 (25)
*S. capitis capitis*	3 (1.4)	3 (100)	1 (33.3)	1 (33,3)
*S. chromogenes*	2 (1.4)	2 (100)	0	0
*S. xylosus*	1 (0.4)	1 (100)	0	0
*S. simulans*	1 (0.4)	1 (100)	0	0
*S. sciuri*	1 (0.4)	1 (100)	0	0
Unidentified CoNS	28 (13.2)	24 (85.7)	2 (7.1)	2 (7,1)
Total	211 (100)	184 (87)	65 (31)	60 (28,4)

*MRD* multidrug resistance, *MIC* minimum inhibitory concentration, *CHX* chlorhexidine, *CoNS* Coagulase-negative staphylococci, *MR* Methicillin-resistant

### Antimicrobial susceptibility

Antibiotic susceptibility testing to the 12 previously mentioned antibiotics revealed that 184/211 (87%) isolates were multidrug resistant. Multidrug resistance was distributed among all species (Table 1). Using the cefoxitin disk, 68.7% of the samples presented resistance, but after detecting the *mecA* gene by PCR, this occurrence increased to 80,5%. Therefore, to 11.3% of the strains, the cefoxitin disk was not as sensitive for detection of oxacillin resistance. Five strains were D-test positive. All samples were sensitive to vancomycin, exhibiting an MIC ≤4 μg/mL [14].

### Minimum inhibitory concentration determination

A total of 65/211 (31%) samples demonstrated reduced susceptibility to chlorhexidine, with MIC values ranging from 4 mg/L to 8 mg/L. Reduced susceptibility to chlorhexidine was observed in several species (Table 1). The vast majority of strains resistant to chlorhexidine also showed multidrug resistance (94%) and cefoxitin resistance (92%).

### Detection of the *qacA/B* gene

The *qacA/B* gene was searched in 64 samples, with 32 resistant to chlorhexidine and 32 susceptibles. All resistant samples were positive for the *qacA/B* gene. On the other hand, this gene was detected in 17 strains susceptible to chlorhexidine. The strains positive for the *qacA/B* gene presented a multidrug resistance profile (82%) and cefoxitin resistance (81%).

## Discussion

In the present study, 211 clinical isolates of coagulase-negative *Staphylococcus* were investigated for susceptibility to antibiotics, decreased phenotypic susceptibility to chlorhexidine, and the presence of *qacA/B* genes, determinants of resistance to chlorhexidine.

The conventional methodology was not adequate to identify all species. This can be explained due to the great diversity of species of CoNS and their variable phenotypic characteristics. *Staphylococcus epidermidis* was most frequently isolated in this study, which agrees with the findings of other studies [20, 21]. *S. auricularis* had a high occurrence, in contrast to other studies [20, 21], which reported a low prevalence. The identification through biochemical methods is still not completely reliable; however, other methodologies such as genotypic methods also have limitations [22, 23].

We observed a difference in the results obtained through the disk diffusion methodology with cefoxitin and by detection of the *mecA* gene. The cefoxitin disk was less sensitive in detectin*g* heterologous populations of oxacillin resistant CoNS; this was also observed in another study carried out in Brazil [24]. The use of the combination of cefoxitin and oxacillin disks may improve the sensitivity of detection of oxacillin resistance in CoNS [25]. This approach is especially useful in laboratories that do not have access to *mecA* PCR, which is the gold standard method for detecting methicillin resistance. Accurate detection of oxacillin resistance is essential because false negative results may bring harm to the patient regarding the drugs of choice in the treatment.

With respect to the evaluation of the susceptibility of chlorhexidine, we observed in the literature that the percentage varies greatly, depending on the region studied, as well as the detection of the *qacA/B* genes [26, 27]. Due to this variability of data and the poorly studied geographic distribution of these genes [3], it is important to monitor the resistance to antiseptics in our territory. Our study verified a number of strains with reduced susceptibility to chlorhexidine similar to that reported in Taiwan with MRSA samples [9]. The identification of CoNS strains is concern since the selective pressure allows these strains to persist in the hospital environment, not only causing infections but also possibly transferring their resistance genes to chlorhexidine to other bacterial pathogens, including *S. aureus*. These results should not be ignored, because may they threaten the effectiveness of prevention and control practices of HAI. Therefore, it is essential that long-term surveillance programs be realized and that the factors related to this event be investigated.

All samples tested with reduced susceptibility to chlorhexidine harbored the *qacA/B* gene. Although other studies have demonstrated a strong relationship between *qacA/B* and reduced susceptibility to chlorhexidine [6, 28], it was observed in this study that the distribution of the *qacA/B* genes is not limited to strains expressing resistance to chlorhexidine. The presence of the *qacA/B* genes does not guarantee gene expression, its expression is influenced by transcription regulators*,* level of exposure and previous contact with cationic agents [16, 29]. Although we had investigated only the *qacA/B* genes, others resistence genes are also associated to biocide reduced susceptibility in staphylococci, including *smr*, *qacH*, *qacJ*, *qacG* and *norA*. Chlorhexidine susceptible strains carrying the *qacA/B* gene may be resistant to other antiseptics, which have not been tested in this study. The correlation between the presence of *qacA/B* and elevated MICs is still not fully understood and it has been demonstrated that the carriage of more than one determinants of resistance concomitantly in an isolate can increase the MIC of chlorhexidine [27].

The study showed that there is antiseptic and antibiotic co-resistance in coagulase-negative staphylococci strains. This was demonstrated by the fact that almost all isolates with reduced susceptibility to chlorhexidine and also displaying multidrug resistance. Furthermore, we detected a high frequency of *qacA/B* positive strains with multidrug resistance, similar to that reported by other authors [4, 17]. Genetic linkage between *qac* genes and genes conferring resistance to erythromycin, trimethoprim, and aminoglycosides on the same Staphylococcal plasmids has been reported [3, 19]. Regarding this, it is believed that the spread of resistance genes to antibiotics may be caused by the selective pressure of frequent use of chlorhexidine [9, 30, 31]. Our results corroborate such studies demonstrating the presence of strains with high pathogenic potential because they are resistant to the two forms of control of HAIs, antiseptics/disinfectants and antibiotics.

## Conclusions

In conclusion, in the present study, 31% of coagulase-negative *Staphylococcus* isolated from clinical samples demonstrated reduction in susceptibility to chlorhexidine. The detection of the *qacA/B* genes was not sufficient to measure the susceptibility to chlorhexidine. The presence of antibiotic and antiseptic co-resistance hinders the elimination of these bacteria from the hospital environment and facilitates the transmission of resistance genes to other bacteria. The investigation of other factors related to the expression of the *qacA/B* gene would help to better understand resistance to chlorhexidine and provide crucial information for the control of HAIs.

## Abbreviations

CLSI: Clinical and Laboratory Standards Institute
CoNS: Coagulase negative staphylococci
HAI: Healthcare-associated Infection
MDR: Multidrug resistant
MIC: Minimum inhibitory concentration
MR: Methicillin-resistant
MRSA: Methicillin-resistant *Staphylococcus aureus*
PCR: Polymerase chain reaction
QAC: Quaternary ammonium compounds
